# Investigating the shape bias in typically developing children and children with autism spectrum disorders

**DOI:** 10.3389/fpsyg.2015.00446

**Published:** 2015-04-21

**Authors:** Emily R. Potrzeba, Deborah Fein, Letitia Naigles

**Affiliations:** ^1^Department of Psychology, University of ConnecticutStorrs, CT, USA; ^2^Department of Allied Health Sciences, University of North Carolina Chapel HillNC, USA

**Keywords:** shape bias, autism, word learning, intermodal preferential looking, developmental disorders

## Abstract

Young typically developing (TD) children have been observed to utilize word learning strategies such as the noun bias and shape bias; these improve their efficiency in acquiring and categorizing novel terms. Children using the shape bias extend object labels to new objects of the same shape; thus, the shape bias prompts the categorization of object words based on the global characteristic of shape over local, discrete details. Individuals with autism spectrum disorders (ASDs) frequently attend to minor details of objects rather than their global structure. Therefore, children with ASD may not use shape bias to acquire new words. Previous research with children with ASD has provided evidence that they parallel TD children in showing a noun bias, but not a shape bias (Tek et al., 2008). However, this sample was small and individual and item differences were not investigated in depth. In an extension of Tek et al. (2008) with twice the sample size and a wider developmental timespan, we tested 32 children with ASD and 35 TD children in a longitudinal study across 20 months using the intermodal preferential looking paradigm. Children saw five triads of novel objects (target, shape-match, color-match) in both NoName and Name trials; those who looked longer at the shape-match during the Name trials than the NoName trials demonstrated a shape bias. The TD group showed a significant shape bias at all visits, beginning at 20 months of age while the language-matched ASD group did not show a significant shape bias at any visit. Within the ASD group, though, some children did show a shape bias; these children had larger vocabularies concurrently and longitudinally. Degree of shape bias elicitation varied by item, but did not seem related to perceptual complexity. We conclude that shape does not appear to be an organizing factor for word learning by children with ASD.

## Introduction

The shape bias is a principle or strategy that children utilize during language acquisition to rapidly learn new nouns. This bias is exhibited when a child extends the name of an object to new objects of the same shape rather than other characteristics such as color or texture (Diesendruck et al., 2003). For example, a child first learning “ball” with reference to a round blue ball would extend that label to other round objects, rather than to other blue objects. The shape bias is robust among typically developing (TD) children older than 18 months or so (Landau et al., 1988; Graham and Poulin-Dubois, 1999; Samuelson and Smith, 2000; Perry and Samuelson, 2011); however, it is not yet clear whether children with neurodevelopmental disorders, such as autism spectrum disorder (ASD), also use the shape bias in word learning. The shape bias has been linked with noun learning (Samuelson, 2002; Perry and Samuelson, 2011); because many children with ASD appear to have little difficulty acquiring a vocabulary of nouns (Eigsti et al., 2007; Swensen et al., 2007), they might also be predicted to show a shape bias. However, the shape bias also requires children to attend to the overall shapes of objects rather than their smaller perceptual details and children with ASD are known to preferentially focus on such details (Happe and Frith, 2006); thus, acquiring a shape bias might be difficult for them (Tek et al., 2008). Furthermore, the ASD population is extremely heterogeneous, with some children apparently developing language typically whereas others manifest language impairments (Tager-Flusberg and Caronna, 2007); therefore, it is possible that a shape bias might be observed in some children with ASD but not others. In the current study, we address both of these issues with a longitudinal investigation of the shape bias in a sample of children with ASD. We address the question of perceptual focus by including stimuli that vary in visual detail and by assessing whether the children focus on overall shape during non-naming as well as naming trials. We address the question of subgroups by including a relatively large (*n* > 30) sample of children with ASD, who vary widely in their language abilities. This large and varied sample, together with the longitudinal design, also allows us to investigate a number of possible relationships between children’s vocabulary size and eventual attainment of a shape bias.

In TD children, the shape bias has been proposed to emerge during the second year of life, in response to their early acquisition of a set of nouns whose referents are objects with differentiated shapes (Smith, 2000; Smith et al., 2002). Support for this proposal comes from studies showing that toddlers who are taught novel nouns with differentiated-shape referents demonstrate a shape bias earlier than children who are taught novel nouns organized by material (Samuelson, 2002; Smith et al., 2002). Moreover, Perry and Samuelson (2011) have recently reported that toddlers who have more words for solid objects organized by shape than for solid objects organized by material show a more consistent shape bias—i.e., the shape bias is seen across more trials. Learning the shape bias seems to have positive consequences for later vocabulary growth, as children who are shown to demonstrate a shape bias at one time point subsequently are reported to have larger vocabularies at later time points (Samuelson and Smith, 2000; Smith et al., 2002). Alternative frameworks have also been proposed, suggesting that the shape bias results from general conceptual mechanisms instead of from the noun-learning process. These frameworks emphasize the function of the creator’s intent for a particular shaped object as the cause for generalization of the name (Diesendruck and Bloom, 2003; Diesendruck et al., 2003).

Especially early in development, children’s demonstration of a shape bias is also influenced by visual properties of the objects themselves. That is, even though object shape is a salient property to preverbal infants (Hupp, 2008), extracting shape similarities across diverse objects is not always a straightforward task. For example, Son et al. (2008) have demonstrated that TD toddlers show a stronger shape bias with perceptually simple objects (e.g., with a smooth shape and a single color) compared with more complex ones (e.g., with more edges and more than one color). Similarly, Tek et al. (2012) found that toddlers extended the labels to new objects more consistently if those new objects matched the original only and exactly in shape, and were paired with objects that matched the original only and exactly in color. Whereas test object pairs that shared some color and shape details with each other were actually more likely to elicit a material bias.

Effects of perceptual detail might be expected to be even stronger in children with ASD, because of their tendency to focus on the small physical details of objects (Happe and Frith, 2006). While enhanced attention to detail can be strength (e.g., Mottron et al., 2006), over-emphasizing small visual details to define objects can hinder children with ASD from noticing the overall shape similarities of those objects. Thus, they might develop a shape bias that is weaker—and/or emerges later—than their TD peers. Consistent with this hypothesis is the common observation that children with ASD manifest delays in the onset of language development; many also show significant impairments in pragmatic abilities and some show grammatical delays or impairments as well (Tager-Flusberg, 2004; Eigsti et al., 2007; Goodwin et al., 2012; Tek et al., 2014). However, researchers have also reported that many children with ASD acquire a substantial vocabulary (Eigsti et al., 2007; Tek et al., 2014). Similarly to TD children, their first words are usually object words, and they demonstrate a noun bias when presented with novel words that could be mapped onto objects or actions (Tager-Flusberg et al., 1990; Swensen et al., 2007). Thus, it is possible that at least some children with ASD have acquired a shape bias for use with learning new words.

To our knowledge, only one published study has investigated the existence of a shape bias in young children with ASD. Tek et al. (2008) examined a group of 15 children with ASD across 12 months of development beginning when they were between 2 and 3 years of age; a TD group (MA = 20 months), which was matched on language to the ASD group at the initial visit, was also tested. The method of assessing language was intermodal preferential looking (IPL; Golinkoff et al., 1987; Naigles and Tovar, 2012), in which children view side-by-side videos and hear a linguistic stimulus that matches only one of the videos. This method has elicited good comprehension of some aspects of language from young children with ASD, partly because it allows them to express their language skills without their social cognitive deficits impeding their performance (Swensen et al., 2007; Naigles et al., 2011; Sasson et al., 2013; Venker et al., 2013).

Indeed, Tek et al. (2008) found that both TD and ASD groups demonstrated usage of a noun bias via the IPL paradigm, in that they preferentially mapped novel words onto novel objects rather than novel actions. Both groups were also tested on the shape bias four times over the course of a year. Beginning at visit 2, when they averaged 24 months of age, the TD children looked significantly longer at the shape-match object during novel-name trials compared with control trials; thus, they demonstrated a shape bias. In contrast, the ASD group did not show the same effects even at the fourth visit, when they averaged 45 months of age and had a lexicon of more than 100 nouns. These children also completed a pointing version of the shape bias task, with 3-dimensional versions of the target and test objects. The pointing task elicited a shape bias from the TD group at 28 and 32 months of age, but no group-wide shape bias was observed from the ASD group at any visit. Thus, the IPL findings replicated those from the pointing task; the earlier demonstration of the shape bias in the TD group via IPL vs. pointing is consistent with other research showing that implicit tasks elicit evidence of linguistic knowledge developmentally earlier than explicit tasks (Hirsh-Pasek and Golinkoff, 1996; Graham and Poulin-Dubois, 1999; Naigles, 2002; Goodwin et al., 2012; Piotroski and Naigles, 2012; Golinkoff et al., 2013). Tek et al. (2008) concluded that these children with ASD did not have a shape bias.

The underlying bases for the absence of a shape bias in children with ASD are still unknown; moreover, this study clearly needs further replication and extension. For one thing, Tek et al.’s (2008) report was from a study still in progress; those children with ASD also viewed the shape bias video at two subsequent visits, when they averaged 49 and 54 months of age. Thus, it is possible that the original study was underpowered, and a reliable shape bias will be seen with more children and/or later in development. Furthermore, while Tek et al. (2008) reported some indications of individual differences, in that children with ASD who had higher vocabulary scores on the MacArthur-Bates Communicative Development Inventory (MB-CDI) showed a stronger shape bias at one visit, they did not investigate the longitudinal antecedents or consequences of an emergent shape bias. Moreover, Tek et al. (2008) did not compare the looking patterns elicited by the different items to see if their perceptual complexity played a role in eliciting a shape bias. In sum, with the current study we address three questions: (1) Will children with ASD demonstrate a shape bias, as a group, with a larger sample size and developmental timespan? Alternatively, will a shape bias be seen consistently in some subgroup(s) of children with ASD? Because the IPL task seems to be more sensitive to the onset of the shape bias (see also Graham and Poulin-Dubois, 1999), we only report IPL findings here. (2) If shape bias performance varies within the ASD group, are their shape-match preferences predicted by their vocabulary size or content, and does their degree of shape-match preference predict later good language skills? (3) Does the perceptual complexity of the individual items play a role in the shape bias performance of the ASD group?

## Materials and Methods

### Participants

Participants for this longitudinal study included 35 TD children (29 male, 6 female) and 32 children with ASD (27 male, 6 female). Participants with ASD resided in Connecticut, Massachusetts, New Jersey, New York, and Rhode Island. Upon beginning the study, the children with ASD’s ages ranged from 24 to 42 months (*M* = 32.8, SD = 5.4). Participants had received a professional diagnosis of ASD within the past 6 months and had begun interventions including 5–30 h per week of applied behavior analysis (ABA) therapy. The diagnosis for each child was confirmed at the first visit.

TD participants resided in the state of Connecticut. Upon beginning the study, their ages ranged from 18 to 23 months (*M* = 20.3, SD = 1.5). Status as a TD participant was also confirmed at the initial visit. Beginning the study, TD and ASD groups did not differ in language or cognitive levels, but were significantly different in adaptive functioning. By visit 6, groups differed significantly in cognitive, language, and adaptive behavior scores (see **Table 1** ). Informed consent was obtained from each child’s parent or guardian at each visit. The University of Connecticut Internal Review Board for human subjects approved all materials and procedures involved in this study.

**Table 1:** **Test scores for both TD and ASD groups**.

		ASD		TD			
		*M*	SD	*M*	SD	*t*	*p*
**Visit 1**
Mullen	Visual	26.91	5.56	26	3.29	0.82	0.415
*raw score*							
	Fine motor	24.7	4.3	22.2	2.12	3.16	0.002^∗^
	Receptive	21.34	9.2	24.08	3.6	-1.63	0.108
	Expressive	17.56	7.34	19.94	5.05	-1.56	0.124
Vineland	Communication	75.41	16.83	104.45	8.91	-8.93	<0.001^∗^
*standard score*							
	Daily Living	77.5	13.96	104.71	8.54	-9.71	<0.001^∗^
	Socialization	73.56	7.53	101.08	6.5	-16.05	<0.001^∗^
	Motor skills	81.43	12.81	100.31	6.64	-7.66	<0.001^∗^
ADOS	Module 1	14.12	3.98	0.82	1.48	18.41	<0.001^∗^
CDI	Infant	84.78	109.33	121.11	109.76	-1.35	0.180
*total produced*							
**Visit 2**
CDI	Toddler	196.12	213.86	329.23	172.7	-2.77	0.007^∗^
*total produced*							
**Visit 3**
CDI	Toddler	229.03	208.66	481.23	145.47	-5.63	<0.001^∗^
*total produced*							
**Visit 4**
CDI	Toddler^a^	116.28	152.19				
*total produced*							
	Level III^b^	59.33	22.46	61.5	24.83	-0.26	0.792
**Visit 5**
ADOS	Module 1^c^	15.26	4.8				
	Module 2^d^	10.83	3.73	1.14	2.20	10.8	<0.001^∗^
CDI	Toddler^e^	105.43	127.54				
*total produced*							
	Level III^f^	61.33	21.91	70.6	20.62	-1.43	0.159
**Visit 6**
Mullen	Visual	38.19	8.26	42.81	8.37	-2.24	0.028^∗^
*raw score*							
	Fine motor	33.06	7.58	37.34	4.02	-2.82	0.006^∗^
	Receptive	31.97	10.47	39.81	4.42	-3.90	<0.001^∗^
	Expressive	27.88	13.26	39.93	5.46	-4.75	<0.001^∗^
Vineland	Communication	85.45	18.74	106.75	10.93	-5.53	<0.001^∗^
*standard score*							
	Daily living	80.09	19.02	103.45	9.91	-6.06	<0.001^∗^
	Socialization	77.13	14.94	101.31	7.94	-8.05	<0.001^∗^
	Motor skills	89.06	15.79	103.37	10.04	-4.31	<0.001^∗^
CDI	Toddler^g^	120.53	131.27				
*total produced*							
	Level III^h^	69.80	22.64	79.67	15.53	-1.73	0.09^∗^

At visits 4–6, the children with ASD were rated on either the CDI-Toddler or CDI-III versions. At visit 5, they participated in either the ADOS-mod 1 or ADOS-mod 2, depending on individual functioning. ^a^n = 18; ^b^n = 12; ^c^n = 20; ^d^n = 12; ^e^n = 16; ^f^n = 15; ^g^n = 13; ^h^n = 15. *p < 0.05.

### Apparatus

The IPL videos were shown to each participant on a large projector screen set up in their home. The child sat approximately four feet in front of the screen; either by themselves, upon a familiar seat of choice, or with a parent or visiting research assistant. Participating parents and research assistants wore headphones playing classical music in order to mask the audio stimuli. A digital camera, focused on the child’s face, was placed centrally below the screen aligned with the child and adjusted for individual height and choice of seating arrangement. The speaker projecting the auditory stimuli was located behind the projection screen and also aligned centrally with the digital camera and child (Naigles and Tovar, 2012).

### Materials

**FIGURE 1:**
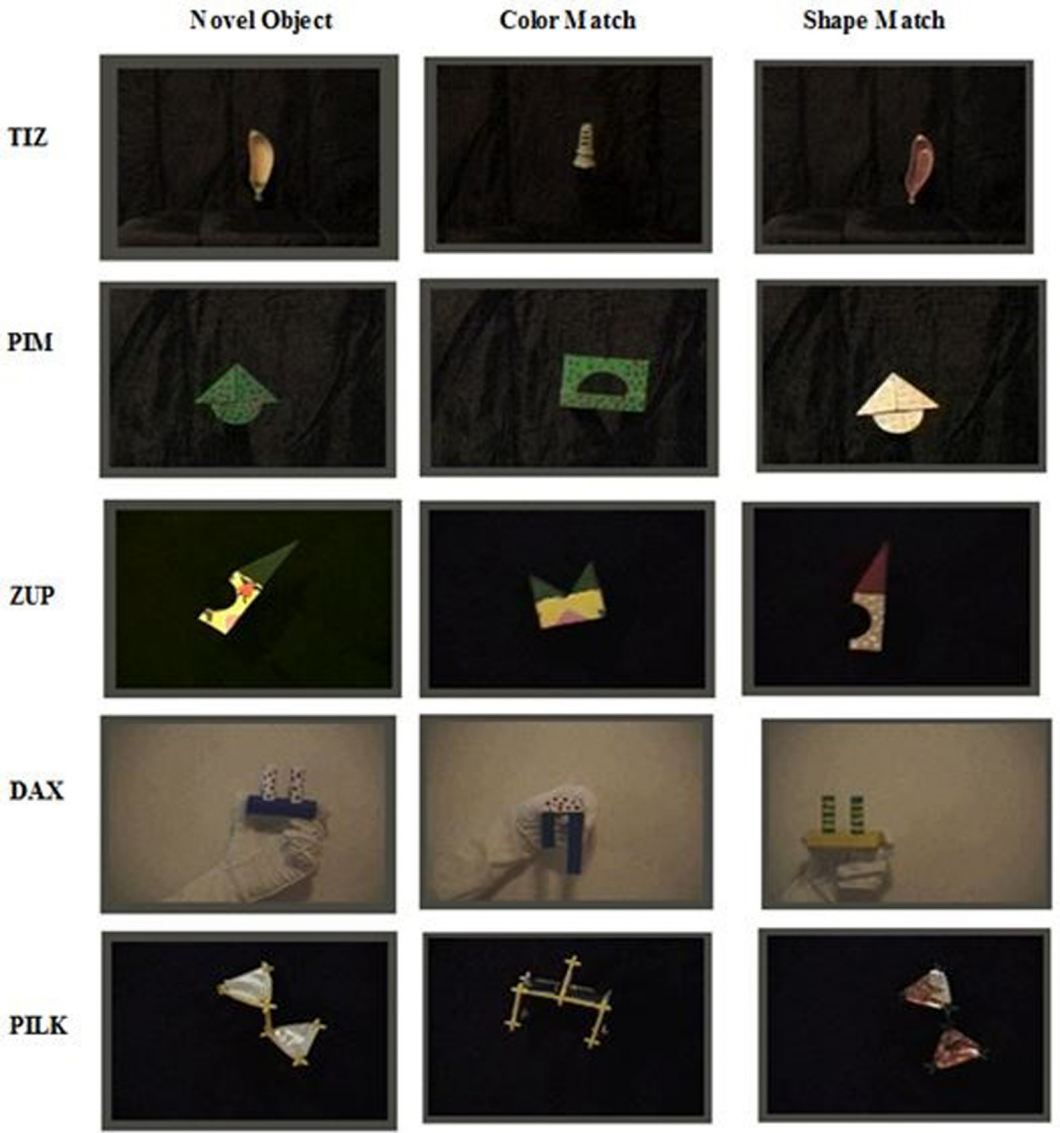
**Novel shape IPL stimuli**.

The shape bias video was the same as that used by Tek et al. (2008). Novel objects were constructed from simple wooden blocks or plastic toys. Wooden blocks were painted with solid, striped, and polka dot design variations. Plastic toys were of unfamiliar shapes and enhanced with decorative paper. Across the objects, the levels of complexity in detail (intuitively operationalized as the number of corners) varied from low to mid to high. Most objects had an element of curvature in their overall structure. In total there were five target objects, five color pattern match objects and five shape-match objects (each color-match and shape-match corresponding with one target). Ordered from lowest to highest complexity of detail, the five novel target objects were labeled Tiz, Pim, Zup, Dax, and Pilk (see **Figure 1**). Each object was filmed moving slowly back and forth; each clip was 4 s long.

The video included a set of five NoName (i.e., control) trials followed by a set of five Name (i.e., test) trials. A sample video layout for one block of NoName and Name trials is shown in **Table 2**; trial 4 is the NoName test and trial 8 is the Name test. During the interstimulus interval, the child was re-centered via a flashing red dot. The side of first presentation of each object varied across objects within the video in an LRLRL pattern, and was counterbalanced between children and across visits (i.e., half of the children viewed variant A at visits 1, 3, and 5 and variant B at visits 2, 4, and 6; the other half experienced the opposite pattern). Variants A and B were also differentiated by the side of presentation of the shape-match, which varied in a LRRLL or RLLRR pattern. When the target was initially presented on either the right or left side of the screen, the opposing side remained black, without a video stimulus. The order in which the objects were presented differed between NoName and Name blocks. The target object did not remain visible on the screen during the simultaneous presentation of color-match and shape-match objects; thus, the children had to remember how it looked during the test trials.

**Table 2:** **Sample of shape bias video layout**.

Video 1 (left side)	Audio	Video 2 (right side)
**NoName block**
(1) Yellow banana boat	Look at this!	Black/no stimulus
(2) Black/no stimulus	Look at this!	Yellow banana boat
(3) Yellow kitchen tool	They’re different now!	Orange banana boat
(4) Yellow kitchen tool	Which one looks the same?	Orange banana boat
**Name block**
(5) Yellow banana boat	Here’s the DAX!	Black/no stimulus
(6) Black/no stimulus	Look, a DAX!	Yellow banana boat
(7) Yellow kitchen tool	They’re different now!	Orange banana boat
(8) Yellow kitchen tool	Where’s the DAX?	Orange banana boat

#### Standardized Test Measures

The Autism Diagnostic Observation Schedule (ADOS; Lord et al., 1989) is a series of structured play activities constructed as a diagnostic assessment of ASDs; this was administered at visits 1 and 5.

The MB-CDI (Fenson et al., 1991) is a parent-report standardized assessment measuring the child’s early language development. There are three versions: Infant, Toddler, and Level III. The CDI Infant version is intended for children ages 8–16 months and measures both language production and comprehension. Part one of this version consists of a 396 word vocabulary inventory including nouns, verbs, adjectives, pronouns, prepositions, and quantifiers. Part two assesses the child’s use of actions and gestures for early non-verbal communication. The CDI Toddler version is intended for children ages 16–30 months. Part one of this version contains a 608 word vocabulary inventory. Part two assesses morphological and syntactic usage. CDI Level III is an 100 word expressive vocabulary inventory with a questionnaire assessing complex semantic, pragmatic, and grammatical usage. For this study only the vocabulary inventories were analyzed. Parents of TD children filled out the Infant version at visit 1, the Toddler version at visits 2 and 3, and Level III at visits 4 through 6. For the children with ASD, the schedule was identical to the TD children for visits 1 through 3. Starting at visit 4, parents of children with ASD filled out the CDI III if their child produced a lexicon of greater than 250 words on the Toddler version. Thus, some children reached Level III at visit 4 and others never advanced past the Toddler version. At visits 4–6 for the ASD group, CDI raw scores were adjusted by calculating the percent of vocabulary items endorsed for each child’s specific checklist; these scores were thus somewhat comparable across the three instruments.

The Vineland Adaptive Behaviors Scales (Sparrow et al., 2005) is a parent-reported questionnaire assessing the child’s developmental milestones across the areas of communication, daily living, socialization, and motor skills. Scores are standardized to compute overall adaptive functioning.

The Mullen Scales of Early Learning (Mullen, 1995) assess the overall intellectual development of the child across the areas of cognition, expressive and receptive language, and motor development. Both raw and standard scores were used in the analyses.

### Procedure

Children were visited in their homes every 4 months for a total of six visits. The first visit was separated into two sessions. At the first session the ADOS, CDI, Vineland, and Mullen were administered and the child was introduced to the IPL paradigm. At the second session, 1 week later, the child was shown the IPL videos. At subsequent visits the IPL videos were presented prior to all other activities.

The shape bias video was shown to each TD participant at visits 1 through 4 and was shown to each participant with ASD at visits 1 through 6. For all participants at visits 1 and 2, the shape bias video was shown as the second of three IPL videos. At visits 3 through 6, the shape bias video was shown first (see Swensen et al., 2007; Naigles et al., 2011; Goodwin et al., 2012; Tovar et al., 2015, for descriptions of the findings from the other videos).

### Coding

#### IPL Coding

The recording of the child’s face was digitized and uploaded to a custom coding program after each visit. During coding, research assistants did not have access to the accompanying auditory stimuli. Each child’s visual fixations were coded frame by frame as right, left, center, or away for all trials. Looking patterns during NoName and Name test trials (e.g., trials 4 and 8 in **Table 2**) were then calculated yielding the primary dependent variable of percent looking to shape-match (i.e., seconds looking to the shape-match divided by total time looking to the shape plus color-matches).The latency of the first look to the shape and color-matches was also calculated but proved uninformative and so will not be considered further (Potrzeba, 2014). For 50% of the participants, multiple research assistants coded the recordings until inter-rater reliability within 0.3 s for each trial was achieved by two coders. For the other participants, inter-rater reliability was assessed for 10% of the data set; correlations between the two coders averaged 0.975 (*p* < 0.0001).

#### Trial and Visit Elimination

For each of the visits, participants’ data were eliminated for a number of reasons. Individual trials with a total looking time of less than 1 s were eliminated because the children’s attention to the stimulus was too brief; these trials were designated as missing and not replaced. Individual participants were eliminated at a given visit if they provided a total of fewer than three paired (i.e., involving the same target item) Name and NoName trials, a side bias of greater than 75% across test trials, or a missed visit. For the ASD group, seven participants were eliminated at visit 1, three participants at visit 2, two participants at visit 3, two participants at visit 4, two participants at visit 5, and one participant at visit 6. For the TD group, 12 participants were eliminated at visit 1, three participants at visit 2, and one participant at visit 3 (see **Table 3**).

**Table 3:** **Number of participants in each group whose data was included at each visit**.

	Visit 1	Visit 2	Visit 3	Visit 4	Visit 5	Visit 6
ASD	*n* = 25	*n* = 29	*n* = 30	*n* = 30	*n* = 30	*n* = 31
TD	*n* = 23	*n* = 32	*n* = 34	*n* = 35		

#### Individual and Item Designations

Children were designated as shape-biased at a given visit if they showed a percentage of looking time to the shape-match of greater than 50% in the Name trial, averaged across items. Correspondingly, they were designated as color-biased at a given visit if they displayed a percentage of looking time to the shape-match lower than 50% of the time during the Name trial, again averaged across items; recalling that if the child has a low percentage of looking to the shape-match, they in turn have a high percentage of looking to the color-match, as uninformative trials where the child was predominantly looking away from the screen and not attending to the items were not used for data analysis.

Children’s looking patterns were also assessed for each item at each visit, as follows: first, each NoName and Name trial was assessed as to whether the percentage of looking time to the shape-match was above or below 50%. Percentages below 50% were designated as “Low” and percentages above 50% were designated as “High.” The shift in percentage from the NoName to the Name trial placed that item particular item for that child at that visit into one of four categories. “Low” for NoName coupled with “High” for Name was designated as ‘shape biased (i.e., LH);’ “High” for NoName coupled with “Low” for Name was designated as ‘color-biased (HL).’ “High” for both a given NoName and Name pairing (HH) indicated an overall shape-match preference, regardless of whether the target had been named, and “Low” for a given NoName and Name pairing (LL) indicated an overall color-match preference, again regardless of whether the target object had been named. The proportion of children who provided LH, HL, HH, and LL patterns was calculated for each item, to investigate whether items varying in perceptual complexity elicited different levels of shape bias.

#### MB-CDI Coding

The Infant and toddler versions of each participant’s MB-CDIs were coded for three subcategories. Following Perry and Samuelson (2011) specific words were designated as shape organized (e.g., *chair, cup*), color organized (e.g., *apple, snow*), or as a descriptive term (e.g., *red, blue*). Apples might seem to be shape-organized, but young children typically experience apples in pieces, such that their color is more salient. For the Infant version, there were a possible total of 84 shape words, 48 color words, and three descriptive words. For the Toddler version, there were a possible 108 shape words, 100 color words, and nine descriptive words. Descriptive words were added to the color category. Totals and percentages were calculated to observe potential predominant word types. Only data from visits 1–3 were included because the CDI-III administered starting at visit 4 did not include enough relevant words.

### Analysis Plan

We first conducted ANOVAs to compare NoName and Name trials collapsed across items, to determine whether the shape bias appeared at any visit for each group. We next explored potential subgroups in shape bias performance, ranging from children always exhibiting the shape bias (i.e., at 100% of the visits) to those rarely exhibiting the shape bias (at 0 visits). Furthermore, we explored which individual differences (e.g., from the standardized test measures) correlated with their shape bias performance and we then examined in detail the extent to which each individual’s particular vocabulary content might have influenced their shape bias performance. Lastly, we examined the whether particular items elicited the shape bias more consistently than others.

## Results

### Group Analyses

The TD group exhibited a consistent increase in percent looking to shape-match during the Name trials compared with the NoName trials, starting as early as 20 months of age; in contrast, the ASD group exhibited no consistent pattern. **Table 4** displays the means and SDs by visit and group. A repeated measure, multivariate ANOVA [2 (group) × 4 (visit) × 2 (trial)] was conducted to compare the groups across NoName and Name trials for visits 1 through 4. A significant effect of trial was obtained [*F*(1,40) = 14.904, *p* < 0.001, η^2^ = 0.271] as well as a significant interaction of trial by group [*F*(1,40) = 4.811, *p* = 0.034, η^2^ = 0.107].

**Table 4:** **Mean proportion looking to shape-match by group**.

	NoName		Name					
	*M*	SD	*M*	SD	*t*	*p*	Cohen’s *d*	Number of nouns on CDI
**TD**
Visit 1	0.49	0.13	0.55	0.08	-1.78	0.044^∗^	0.55	>100
Visit 2	0.47	0.11	0.55	0.13	-2.75	0.005^∗^	0.66	>100
Visit 3	0.49	0.08	0.57	0.11	-3.61	0.0005^∗^	0.83	>100
Visit 4	0.51	0.08	0.57	0.08	-3.31	0.001^∗^	0.75	>100
**ASD**
Visit 1	0.52	0.16	0.51	0.14	0.303	0.3825	0.06	<100
Visit 2	0.53	0.11	0.55	0.15	-0.56	0.2905	0.15	>100
Visit 3	0.53	0.1	0.53	0.11	-0.28	0.388	0	>100
Visit 4	0.49	0.07	0.52	0.09	-1.01	0.1615	0.37	>100
Visit 5	0.49	0.09	0.50	0.13	-0.39	0.349	0.09	>100
Visit 6	0.53	0.09	0.49	0.12	1.52	0.0705	0.38	>100

*p < 0.05.

Two additional repeated measures multivariate ANOVAs were then conducted to assess each group separately. For the TD group, the analysis was conducted across visits 1 through 4. A significant effect of trial was found [*F*(1,20) = 19.885, *p* < 0.001, η^2^ = 0.499] and no other significant effects or interactions. Paired sample *t*-tests of the TD children demonstrated a significantly greater percent looking to the shape-match during the Name than the NoName trials at each visit (see **Table 4**; one-tailed tests are reported because the prediction is for greater looking to the shape-match during the Name trials). The effect sizes for the TD group are at similar levels to those reported in other IPL studies (e.g., Gertner et al., 2006; Wagner et al., 2009; Golinkoff et al., 2013). For the ASD group, the analysis was conducted across all six visits; no significant effects or interactions were observed. Paired sample one-tailed *t*-tests comparing the NoName and Name trials were performed but none yielded significant effects (*p*s > 0.14). These analyses were repeated including the children’s percent looking to shape-match during only the first or second halves of the test trials, with similar results.

Children in the TD and ASD groups were then assigned to one of four subgroups, according to the percent of visits for which they showed a shape bias (i.e., looked longer at the shape-match during Name compared to NoName trials). Children in the Always subgroup showed a shape bias at 100% of their visits, children in the Consistent group showed a shape bias at 60–95% of their visits, children in the Inconsistent group showed a shape bias at 40–55% of their visits, and children in the Rarely group showed a shape bias at 0–35% of their visits. The majority of children in the TD group demonstrated a shape bias at more than half of their visits (see **Table 5**); in contrast performance in the ASD group was much more variable. Three children with ASD showed a shape bias at 100% of their visits; however, the majority of children with ASD showed a shape bias at fewer than 50% of their visits (see **Table 5**). A chi-square analysis revealed that the distributions of the two groups were significantly different [χ(3) = 13.6, *p* = 0.003].

**Table 5:** **Number of children in each subgroup of shape bias performance**.

	Always	Consistent	Inconsistent	Rarely
TD	13	15	2	5
ASD	3	10	10	9

*Always = Children who demonstrated a shape bias with all visits. Consistent = Children who demonstrated a shape bias 60–90% of visits. Inconsistent = Children who demonstrated a shape bias at 40–55% of visits. Rarely = Children who demonstrated a shape bias at 0–35% of visits.*

### Individual Differences

Correlations were conducted to investigate the relationships between the children’s standardized test scores, including the MB-CDI, Mullen, and Vineland, and their degree of shape bias (i.e., mean difference of percent looking to shape-match between NoName and Name trials) at each visit. No significant concurrent correlations emerged for the TD group, likely because of little variance in shape bias performance. However, for the ASD group significant concurrent correlations emerged at both visit 2 and visit 6. At visit 2, children’s degree of shape bias positively correlated with their MB-CDI scores (*r* = 0.452, *p* = 0.014). At visit 6, their degree of shape bias positively correlated with their Vineland motor scores (*r* = 0.386, *p* = 0.035), Mullen fine motor raw scores (*r* = 0.363, *p* = 0.045), and Mullen receptive language raw scores (*r* = 0.359, *p* = 0.047). At both early and later visits, then, children with ASD with stronger shape biases had more advanced language skills. Furthermore, at the last visit children with ASD showing the shape bias also had stronger motor skills.

Cross-visit correlations were then conducted between the children with ASD’s MB-CDI scores and their shape bias performance. Four significant relationships were observed: children’s vocabulary at visit 1 correlated significantly and positively with their shape bias performance at visits 2 (*r* = 0.499, *p* = 0.006) and 6 (*r* = 0.409, *p* = 0.022), children’s vocabulary at visit 2 correlated significantly and positively with their shape bias performance at visit 6 (*r* = 0.364, *p* = 0.048), and children’s shape bias performance at visit 4 correlated significantly and positively with their vocabulary at visit 6 (*r* = 0.409, *p* = 0.034).

Multiple regressions were then performed, to investigate whether the earlier vocabulary measures predicted later shape bias performance when controlling for early shape bias performance, and to investigate whether early shape bias performance predicted later vocabulary, when controlling for early vocabulary. Three models were significant: MB-CDI at visit 1 significantly predicted shape bias performance at visit 2 (ΔR2 = 0.218, β = 0.459, *p* = 0.025); shape bias performance at visit 1 did not contribute significantly to the model. Similarly, MB-CDI at visit 1 significantly predicted shape bias performance at visit 6 (ΔR2 = 0.214, β = 0.462, *p* = 0.023); again, shape bias performance at visit 1 did not contribute significantly to the model. Finally, shape bias performance at visit 4 significantly predicted MB-CDI levels at visit 6 (ΔR2 = 0.055, β = 0.237, *p* = 0.033), with vocabulary at visit 4 contributed significantly and independently to this model (ΔR2 = 0.702, β = 0.798, *p* < 0.001). In sum, there seems to be a longitudinal and mutually facilitative connection between vocabulary size and shape bias performance, as children with ASD who had larger vocabularies at the early visits showed a stronger shape bias at one of the later visits, and children who showed a stronger shape bias in the middle of the study were reported to have a larger vocabulary at the last visit.

We further explored this connection between vocabulary and the shape bias by considering whether learning a ‘threshold number’ of shape words were necessary to abstract the shape bias. If this was the case, then children who showed the shape bias more consistently should produce relatively more ‘shape’ words than children who showed the shape bias less consistently. **Table 6** presents the mean percentages of ‘shape’ and ‘color’ words produced at visits 1, 2, and 3, organized by the shape bias subgroups, for the children with ASD. Because of the low number of children in the Always subgroup, the Always and Consistent children were combined into one subgroup for this analysis. As **Table 6** shows, in general, children produced a greater proportion of words in the ‘shape’ category than in the ‘color’ category; moreover, the children who showed the shape bias more consistently produced a greater proportion of words than the children who demonstrated the shape bias less consistently. However, no differences in the distribution of ‘shape’ vs. ‘color’ words were observed at any visit (all chi-squares < 1). That is, the higher proportion of ‘shape’ words produced by the Always/Consistent subgroup is mirrored by the higher proportion of ‘color’ words they produced. In other words, as also demonstrated by the correlation and regression analyses, children with more consistent shape bias performances produced more words overall; there is little indication of a special role for their ‘shape’ words.

**Table 6:** **Mean percent of words in CDI category produced by children with ASD in each shape bias subgroup**.

	Shape bias subgroup		
*CDI Category*	Always/consistent	Inconsistent	Rarely
**Visit 1**
Shape	32.69	19.9	8.89
Color	16.23	8.90	3.67
**Visit 2**
Shape	55.75	48.00	18.25
Color	44.58	33.10	13.00
**Visit 3**
Shape	59.27	56.90	21.14
Color	45.73	41.30	18.00

### Item Effects

**FIGURE 2:**
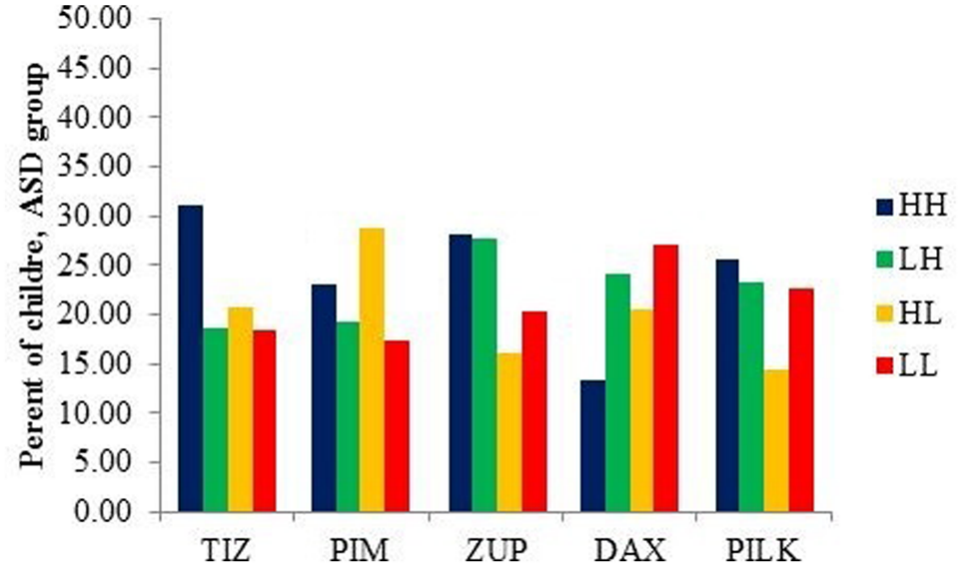
**Percentage of children with ASD showing each pattern of looking for each item (collapsed across visits)**.

Finally, we investigated the degree of shape- vs. color- preferences elicited by each item, for the ASD group. **Figure 2** shows the percent of children with ASD who demonstrated the four looking patterns (LH, HL, HH, LL) for each item, combined across visits. The items are ordered left to right from simplest (fewest corners, tiz) to most complex (most corners, pilk). The items do seem to vary in the type of looking pattern they most commonly elicit, and this variability is confirmed by a significant chi-square analysis [χ^2^(12) = 28.9, *p* = 0.004]. However, the overall pattern is rather complex: if the dominant basis for eliciting a shape bias—or overall shape preference—were perceptual complexity, operationalized here by the number of corners on the object, then the green and blue bars would be highest for TIZ while the orange and red bars (LL, HL) would be highest for PILK. However, while ZUP, a 6-cornered object, clearly elicited more shape-match preferences and DAX, an 8-cornered object, tended to elicit more color-match preferences, the other objects do not fit into either a shape-oriented or a color-oriented pattern.

## Discussion

Children in this study saw triads of novel objects (target, shape-match, color-match) in both NoName and Name trials; those who looked longer at the shape-match during the Name trials than the NoName trials demonstrated a shape bias. Target objects did not remain visible during the presentation of shape or color-match objects; thus, a memory constraint was imposed. Children were tested across four (TD group) or six (ASD group) visits, 4 months apart. The TD group showed a significant shape bias at all visits, beginning at 20 months of age. The ASD group did not show a significant shape bias at any visit, even as late as 54 months of age. Considerable individual variation was observed, however, with slightly more than one-third of the sample demonstrating a shape bias at more than half of their visits, and slightly less than one-third demonstrating a shape bias at 2 or fewer visits. Children with ASD who had larger vocabularies showed a stronger shape bias both concurrently and longitudinally; moreover, children with ASD with a stronger shape bias at visit 4 had larger vocabularies at later visits. Finally, while the target objects varied in perceptual complexity and in the degree to which they elicited a shape bias from children with ASD, there was little indication that these two types of variance were related to each other. Taken together, these findings shed new light on the universality and underlying basis of the shape bias in young children.

Our demonstration of a shape bias in TD children as young as 20 months of age replicates many others in the field (Smith, 2000; Perry and Samuelson, 2011, passim): from midway through the second year of life through 2.5 years of age, children showing typical development preferentially extend the labels of objects to new instances of the same shape rather than color or pattern. Tek et al. (2008), using these same stimuli, had only found a shape bias in TD children by 24 months of age; however, their sample size was small. In this study we doubled the sample size and obtained a significant shape bias in the youngest group tested, indicating that the previous null effect was likely attributable to low power. Nonetheless, the effect size of the 20 month-olds was smaller than that of the older children, indicating that the shape bias increases in strength across this period of development.

In contrast, doubling the sample size for the ASD group, as well as extending the age range, did not change the effects reported by Tek et al. (2008). As a group, the children with ASD did not exhibit a preference for the shape-match during the Name trials compared with the NoName trials. In fact, the children with ASD appeared to look randomly during both the NoName and Name trials; that is, they looked preferentially neither at the shape- nor color-matched objects, both when the target object had been named and when it had not. Thus, while they were not disposed—as a group—to sort the objects by shape, neither were they disposed to do so by color or pattern. This negative finding contrasts somewhat puzzlingly with the positive findings reported for this same sample of children during their other IPL tasks. That is, as a group, these children understand SVO word order and can learn novel verbs using Syntactic Bootstrapping (Naigles et al., 2011); they manifest a noun bias, mapping novel words onto novel objects rather than actions (Tek et al., 2008), and the majority of them also demonstrated understanding of subject- and object-wh- questions, as well as the –ing/-ed aspectual distinction, by visit 6 (Goodwin et al., 2012; Tovar et al., 2015). Thus, their difficulty as a group with the shape bias cannot be attributed to difficulties with the IPL tasks nor with general language comprehension.

However, poor shape bias performance was not universal in our ASD sample, as 13 of the 32 children with ASD did demonstrate a shape bias during more than half of their visits. Many of these 13 were indeed high-functioning (Tek et al., 2013); however, two others were actually non-verbal and three were verbal but still quite delayed in their language development. And five children who had been designated as high-verbal showed a shape bias at fewer than half of their visits. Thus, whereas Samuelson and smith (1999) and Perry and Samuelson (2011) have shown that among TD toddlers, a threshold level of 100 count nouns and/or some number of ‘shape’ words is associated with a shape bias, we did not observe such a threshold level for the ASD sample. Nonetheless, across the entire sample, children with ASD who had higher vocabulary scores, especially at visits 2 and 6, showed a stronger shape bias at the same visit. These findings replicate those from much younger TD children, showing that the shape bias is associated with overall vocabulary size (Samuelson and smith, 1999). Moreover, our findings from the ASD group also replicated those involving TD children with respect to longer-term antecedents and consequences of shape bias performance. That is, Smith et al. (2002) and Smith and Samuelson (2006) have reported that children who develop a shape bias earlier in their second year have larger vocabularies during their third year, controlling for variation in vocabulary size at the initial time point. A similar relationship was observed in our ASD group, where children with a stronger shape bias at visit 4 were reported to produce a greater proportion of the available words on the MB-CDI 8 months later, controlling for their MB-CDI scores at visit 4. It is possible that, the more a child can use the shape bias strategy, the more words they are able to learn. The reciprocal relationship was also observed in our ASD group, that children producing more words on the MB-CDI at visit 1 showed a stronger shape bias at visits 2 and 6, controlling for their degree of shape bias at visit 1. That is, an early demonstrated ability to learn words evidently facilitates the later development of the shape bias. These relationships were observed only in our ASD group, possibly because the TD group was already showing a consistent shape bias at visit 1, and demonstrated much less within-group variability.

Interestingly, at visit 6 children with ASD with stronger shape biases also had higher fine motor scores, judged by both parent report and administered tests. A relationship between the shape bias and motor ability has not previously been reported in the TD literature; however, we conjecture that this might be attributed to the children with ASD’s developing facility with object manipulation. It seems likely, for example, that children who are becoming skillful at manipulating objects might be better at extracting these objects’ global shape characteristics, which might then transfer to the visual extraction of shape in the IPL task. Furthermore, as a result of more skillful manipulation, it is possible that the children can appraise more meaning and functionality to the object, which would allow for broader shape understanding.

Increasing the ASD sample size, then, was fruitful for illuminating how child-based constructs such as vocabulary size and motor skills might influence, and/or be influenced by, the development of a shape bias in children with ASD. In contrast, our second goal of shedding light on the role of object complexity did not bear much fruit. While our five objects varied in perceptual complexity as well as in degree of shape bias elicitation, these two properties did not seem to be related. Our study was limited in that we included only five objects, whose perceptual properties were not varied systematically. Including more objects, though, would have lengthened the video and so further tested the attention spans of the children with ASD.

In sum, one of the same factors that influence the development of the shape bias in TD children—vocabulary size—also seems to influence shape bias performance in children with ASD. This finding supports the universality of the role of the lexicon in the development of this construct. However, the current study also demonstrates that simple objects and sizeable ‘shape word’ vocabularies are not sufficient for children to demonstrate a shape bias, because most of our child participants with ASD displayed such a bias only inconsistently. So why might the shape bias be so challenging for children with ASD? One possibility is that our IPL task imposed more strenuous memory demands than the usual pointing tasks, because in the latter tasks the target object is still available during test. However, both the noun bias and syntactic bootstrapping videos placed similar memory constraints on these children, but for these latter videos the children with ASD were able to succeed, in that they demonstrated consistent looking at the same test stimulus as the TD children (Tek et al., 2008; Naigles et al., 2011).

Another possibility is that the shape bias actually requires more conceptual knowledge than theorists such as Smith (2000) have proposed. For example, Diesendruck et al. (2003) have suggested that children extend object kinds by shape based on the object creator’s intentions; therefore, children with ASD’s difficulties with the shape bias might be related to their well-known difficulties with understanding the intentions of others (Diesendruck et al., 2003). Along similar lines, the lack of shape bias in children with ASD might be consistent with—and possibly symptomatic of—additional difficulties with categorization and lexical organization that have been reported for this population (Minshew et al., 2002; Dunn and Bates, 2005; Gastgeb et al., 2006). That is, the shape bias requires children to utilize words as indicators of category structure (i.e., that different objects are exemplars of the same category), and research with older children with ASD has demonstrated weaknesses and inconsistencies in their category structure (Naigles et al., 2013). A future direction for our research will be investigate the degree to which individual variation in shape bias performance during ages 2–4 is related to variation in category knowledge during school age and adolescence.

Limitations of this study include, as stated above, the lack of systematicity in the investigation of the role of perceptual complexity in developing a shape bias. Moreover, the heterogeneity of our ASD sample may limit generalization of these findings to other populations. Furthermore, it should be noted that this study was conducted with a particularly structured methodology which may not yield findings generalizable across variations of stimuli or across more naturalistic settings. And unlike (Tek et al., 2008), we did not compare preference to the shape-match between looking time via the IPL paradigm and pointing with a hands-on physical object manipulation task; such a comparison could be valuable in future research.

It is understood that the shape bias is a beneficial mechanism for language development and it could become a critical target for early intervention in children with ASD. Future research should aim to further differentiate between the children with ASD who do and do not exhibit the shape bias. Perhaps these children without the bias are not receiving adequate input as pertaining to shape organization and need to be more explicitly taught. Continuing this investigation will yield more knowledge as to the irregularities displayed during language acquisition in children with autism.

## Conflict of Interest Statement

The authors declare that the research was conducted in the absence of any commercial or financial relationships that could be construed as a potential conflict of interest.
